# The Effects and Molecular Mechanisms of a Peptide from *Periplaneta americana* L. in Skin Wound Healing

**DOI:** 10.3390/molecules31081355

**Published:** 2026-04-21

**Authors:** Honghong Qiu, Yanyan Chen, Wei Zhang, Bin Dong, Dongli Zhang, Renjin Tang, Zhong Liu

**Affiliations:** 1 Guangdong Provincial Key Laboratory of Bioengineering Medicine, National Engineering Research Center of Genetic Medicine, Institute of Biomedicine, College of Life Science and Technology, Jinan University, Guangzhou 510632, China; 2 College of Bee Science and Biomedicine, Fujian Agriculture and Forestry University, Fuzhou 350002, China; 3 Hybio Pharmaceutical Co., Ltd., Shenzhen 518000, China; 4 Key Laboratory of Innovative Technologies for Natural Product Cosmetics Raw Materials, Guangdong Provincial Medical Products Administration, Guangzhou 510632, China; 5 State Key Laboratory of Mechanism and Quality of Chinese Medicine, Macau University of Science and Technology, Macau 999078, China

**Keywords:** peptide, *Periplaneta americana* L., skin wound healing, EGFR, HaCaT

## Abstract

*Periplaneta americana* extract can promote wound healing and may play an important role in skin wound healing. In this study, we identified a peptide (DL-13) from *Periplaneta americana* L. and explored its role and mechanisms in skin wound healing. In vitro, the effects of DL-13 on proliferation, migration, and related gene/protein expression in HaCaT keratinocytes were assessed via qRT-PCR and Western blot. In vivo, rat wound healing assays confirmed its efficacy. Results showed DL-13 accelerated rat wound healing. In in vitro studies, DL-13 activated EGFR and its downstream PI3K/AKT/mTOR, ERK/MAPK, and JAK2/STAT3 pathways, upregulated EMT-related proteins (N-cadherin, MMP-2, p-FAK, β-catenin), partially regulated macrophage cytokine secretion, and promoted HaCaT proliferation/migration, thereby facilitating re-epithelialization at skin injury sites. Overall, DL-13 may enhance the function of HaCaT cells by activating the EGFR signaling pathway and regulate inflammatory factors in macrophages, thereby promoting the healing of skin wounds in rats. The results of this study will lay an experimental and scientific foundation for the discovery of new compounds for wound healing and their application.

## 1. Introduction

The skin, as the body’s primary interface with the external environment, serves as a sophisticated biological barrier that protects against pathogen invasion, physical trauma, chemical exposure, ultraviolet (UV) radiation, and fluid loss [1,2,3]. This multilayered organ maintains homeostasis through thermoregulation, sensory perception, and metabolic stability while safeguarding internal tissues from environmental stressors. Cutaneous injury occurs when mechanical or chemical forces disrupt skin integrity, creating tissue defects requiring coordinated repair [4,5]. The wound healing process comprises four distinct yet interconnected phases: hemostasis, inflammation, proliferation, and remodeling [6,7,8,9]. Current therapeutic development faces challenges in clinical validation and economic viability, though several targeted agents addressing critical repair mechanisms (wound debridement, angiogenesis, and cellular proliferation) have demonstrated clinical efficacy [10,11,12,13]. Approaches to treating chronic wounds including cell therapies that harness the regenerative potential of living cells, such as leukocyte- and platelet-rich fibrin (L-PRF), have emerged as promising alternatives that promote wound healing [14,15,16,17,18]. L-PRF contains a high concentration of growth factors and cytokines, which can stimulate cell proliferation, migration, and angiogenesis [19,20,21]. Immunotherapy, on the other hand, uses growth factors or cytokines to regulate the immune response at the wound site to promote healing and reduce inflammation. As for the therapies currently available, only a handful are Food and Drug Administration (FDA) approved, and all are cell-based [22]. Therefore, traditional wound treatment remains essential for managing acute wounds and preventing infections. Despite growing interest in bioactive peptides for their stability and cost-effectiveness, few candidates have progressed to clinical application.

*Periplaneta americana* L., an evolutionarily resilient insect species, has emerged as a valuable resource in pharmacological research due to its diverse bioactive compounds [23]. In recent years, with the deepening of research on the biological activity and related preparations of *Periplaneta americana* L., it has been found that its pharmacological effects are mainly concentrated in anti-tumor activity [24], wound healing and tissue repair [25], as well as antibacterial [26] and anti-inflammatory effects [27]. Isolated bioactive components, including ethanol extract (e.g., Kangfuxin), protein–polysaccharide complexes (PaPPc2, PaPPc3), glycoproteins (PAGP-1, PAGP-2), low-molecular-weight mixed peptides (<3 kDa), and exosome-like nanoparticles (PA-ELNs), have been validated to promote skin and mucosal repair. Mechanisms involve enhancing keratinocyte/fibroblast proliferation and migration, increasing EGF/VEGF secretion, regulating inflammatory factor release, and accelerating collagen synthesis [25,28,29,30,31,32]. Additionally, its extracts can repair colonic mucosal lesions, demonstrating multi-site repair potential [33].

However, the current research has notable limitations: first, most studies focus on crude extracts or complex fractions, which have complex compositions and quality control challenges, while the isolation and identification of individual core bioactive peptides remain insufficient; second, reported low-molecular-weight peptides are predominantly mixed fractions, lacking systematic characterization of individual peptides; third, no prior study has reported the single peptide “DL-13”, and its novelty as a *P. americana*-derived component, and structural/functional differences from known fractions remain unaddressed. We have applied for a patent for this peptide [34], with the patent number being CN 106977586 A. The sequence is H-Ala-Ala-Pro-Pro-Ser-Asn-Leu-Lys-Glu-Val-Pro-Ile-Ile-Ala-Tyr-OH, and the structural characteristics of the peptide are all described in the patent. Therefore, this study aimed to evaluate the skin wound healing promoting action and mechanism of a novel peptide, DL-13, derived from *Periplaneta americana* L., which may provide the possibility that DL-13 has strong potential as a wound repair drug candidate.

## 2. Results

### 2.1. Chromatographic Separation of DL-13

**Figure 1:**
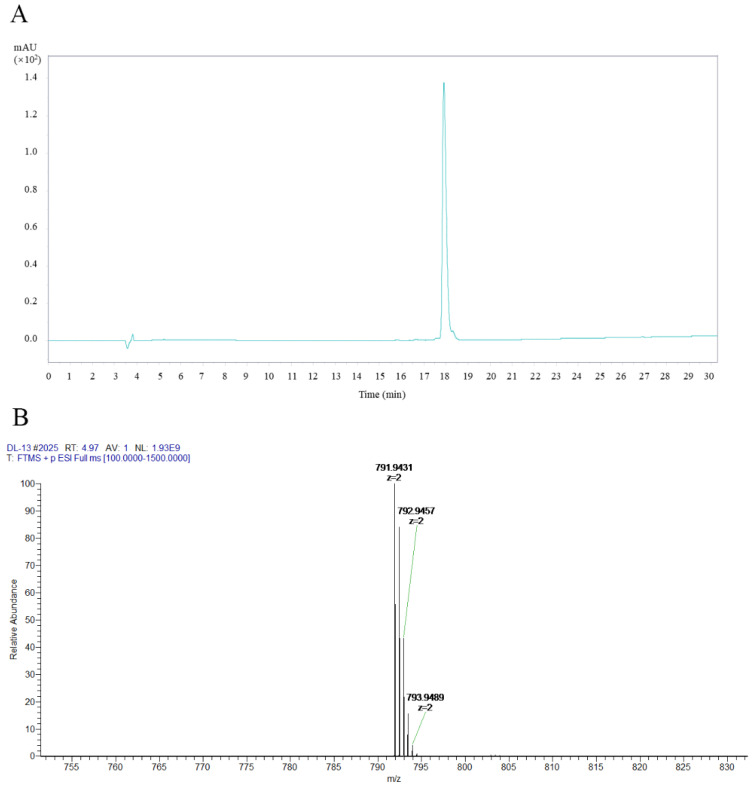
Chromatographic separation of DL-13. (**A**) HPLC profile of DL-13; (**B**) HR-ESI-MS analysis of DL-13.

The purity of DL-13 was confirmed to exceed 95% by analytical high-performance liquid chromatography (HPLC) (Figure 1A), and its molecular weight was determined by mass spectrometry. High-Resolution Electrospray Ionization–Mass Spectrometry (HR-ESI-MS) displayed a doubly charged ion peak at *m*/*z* 791.9431 [M+2H]^2+^, corresponding to a molecular weight of 1582.8839 Da.

### 2.2. Effect of DL-13 on Viability of HaCaT Cells

**Figure 2:**
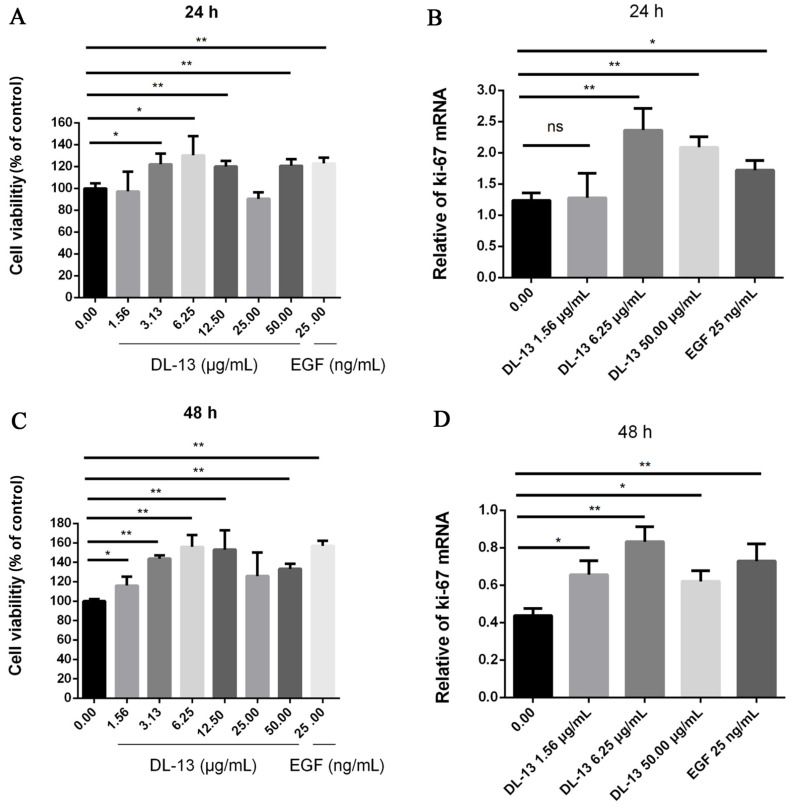
HaCaT cell viability test. MTT was used to test the viability in different groups for (**A**) 24 h and (**C**) 48 h, and the results were detected by spectrophotometer. EGF is a potent positive control used at standard nanomolar concentrations (ng/mL), whereas DL-13 acts as a peptide drug candidate requiring micromolar concentrations (μg/mL). Vertical axis, absorbance; horizontal axis, time. Detection of cell proliferation ability by the Ki-67 test. The expression of Ki-67 in HaCaT cells was detected after treatment with DL-13 for (**B**) 24 h and (**D**) 48 h. Data are shown as mean ± SEM (* *p* < 0.05; ** *p* < 0.01; ns indicates no significant difference (*p* ≥ 0.05)).

The methylthiazolyldiphenyl-tetrazolium bromide (MTT) assay was adapted from the classic protocol reported by Mosmann [35] and optimized for HaCaT keratinocytes. We found that DL-13 did not induce cell death within 24 h and 48 h until increasing the dose to 50 μg/mL. Cell proliferation is essential for the recovery of skin integrity [36] through the migration and proliferation of the epidermal cells from the wound edges to accelerate the epithelium of the trauma site [37,38]. Compared with the blank control group, DL-13 significantly stimulated the proliferation of human immortalized keratinocytes (HaCaTs) in a dose-dependent manner up to 6.25 μg/mL, at which the optimal effect was observed (Figure 2A,C). The qPCR protocol was adapted from the standard workflow described by Bustin et al. [39]. Likewise, data from the mRNA level of Ki-67 (Ki-67) by Quantitative Real-time Polymerase Chain Reaction (qPCR) revealed that 6.25 μg/mL DL-13 promoted cell proliferation within 24 h and 48 h (Figure 2B,D).

### 2.3. Effect of DL-13 on Migration of HaCaT Cells

**Figure 3:**
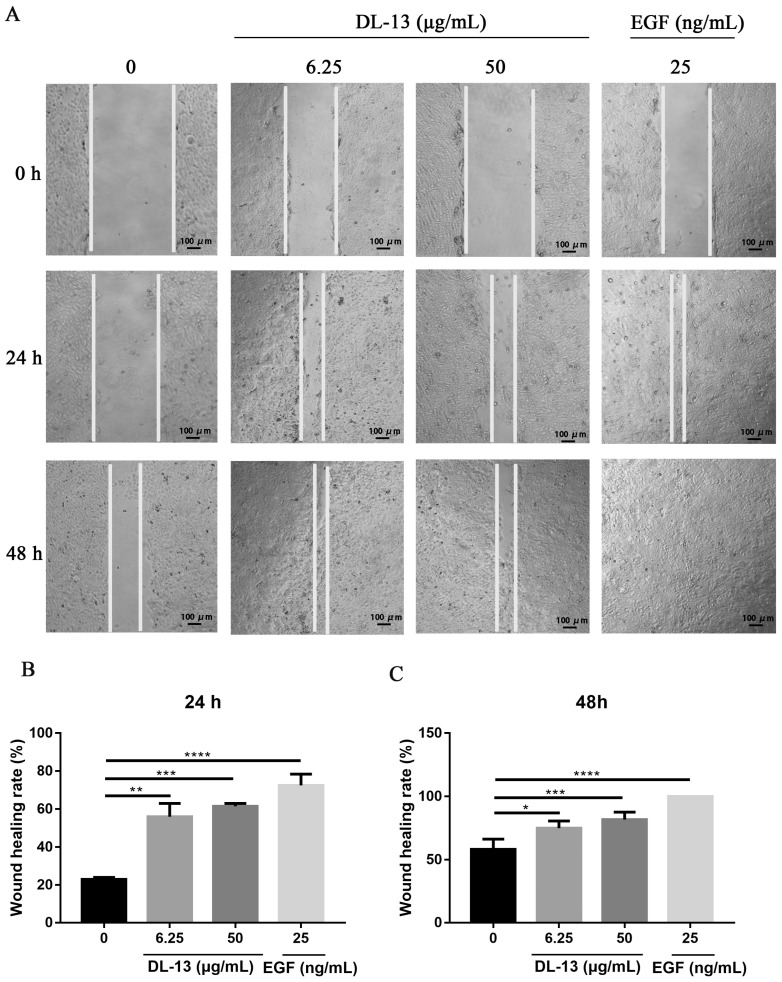
Migration experiment of HaCaT cells. (**A**) The results of the cell scratch test were photographed by light microscope. (**B**,**C**) The area of the uncured scratch was calculated by ImageJ. Data are shown as mean ± SEM (* *p* < 0.05; ** *p* < 0.01; *** *p* < 0.001; **** *p* < 0.0001).

The effects of DL-13 on HaCaT cell migration were determined using the scratch assay. DL-13 could promote cell migration of HaCaT cells at concentrations of 6.25 and 50 μg/mL in 24 h and 48 h (Figure 3A). The quantification of the migration rate is shown in Figure 3B. The average migration rates of the DL-13 groups at 6.25 and 50 μg/mL were 56% and 61.5% after 24 h and that of the blank group was 23%. The average migration rates of the DL-13 groups at 6.25 and 50 μg/mL were 75% and 81.75% after 48 h and that of the blank group was 58.3% (Figure 3C). It was demonstrated that DL-13 showed a saturated stimulation to the migration of HaCaT cells in 24 h.

### 2.4. Effect of DL-13 on Inflammatory Cytokines in LPS-Induced Inflammatory Cell Models

**Figure 4:**
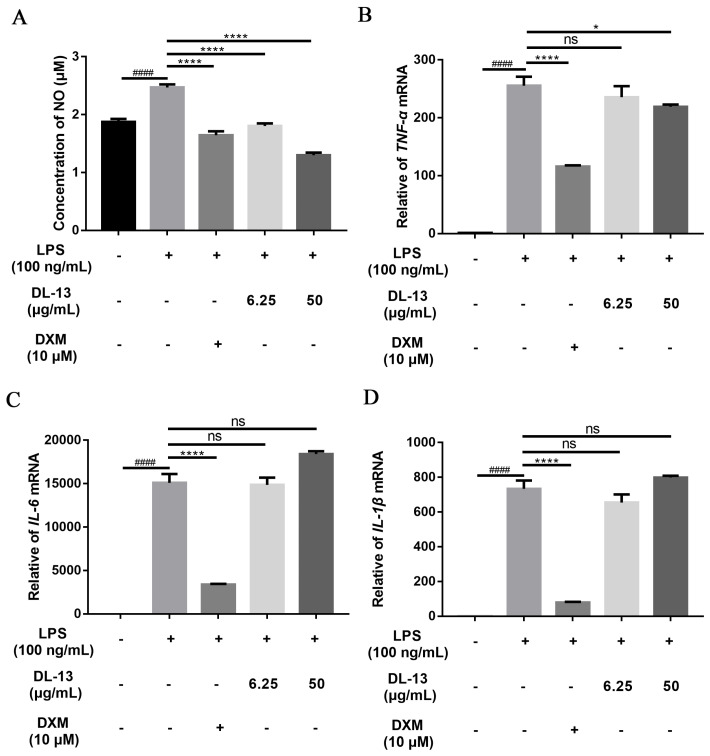
Effect of *Periplaneta americana* DL-13 on LPS-induced inflammatory cytokine expression in Raw 264.7 cells. DXM as dexamethasone (the positive control). (**A**) NO; (**B**) *TNF-α*; (**C**) *IL-6*; (**D**) *IL-1β*. Data are shown as mean ± SEM (^####^ *p* < 0.0001, compared with the control, * *p* < 0.05; **** *p* < 0.0001; ns indicates no significant difference (*p* ≥ 0.05), compared with the LPS-only group).

To define the targets of DL-13 involved in the inflammatory status, the expression of nitric oxide (NO) was evaluated, while the gene expressions of tumor necrosis factor-alpha (TNF-α), interleukin-6 (IL-6), and interleukin-1β (IL-1β) were evaluated by qPCR. The mRNA level of *TNFα* and the secretion level of NO were decreased by DL-13 in a concentration-dependent manner (Figure 4A,B). We found that DL-13 treatment did not decrease *IL-6* and *IL-1β* gene expressions (Figure 4C,D). These results suggest that DL-13 exerts an inhibitory effect on the inflammatory response, potentially by regulating *TNF-α* expression.

### 2.5. DL-13 Could Activate EGFR and Its Downstream ERK/MAPK and JAK/STAT3 Signaling Pathways and mTOR Signaling Pathway-Related Signaling Molecules as Well as EMT Pathways

**Figure 5:**
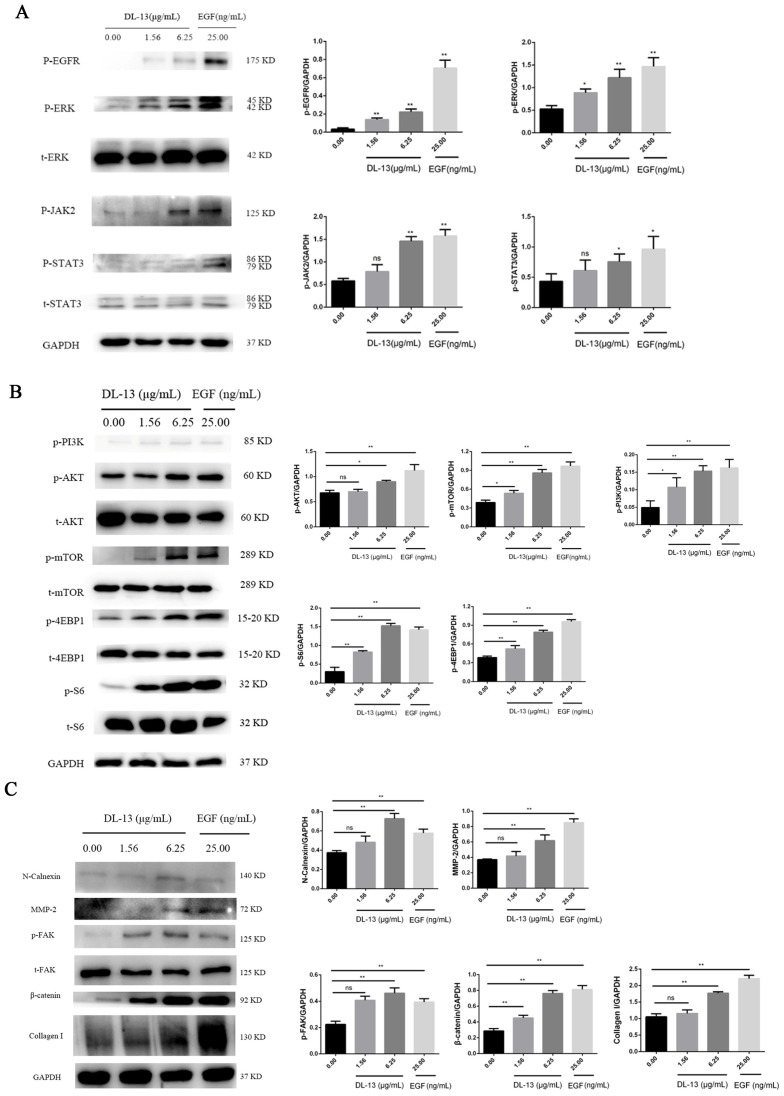
The changes in pathway-related signaling molecules after DL-13 treatment. DL-13 was used to stimulate the cells in vitro, and the cell proteins were extracted. (**A**) The expressions of EGFR, ERK, JAK2, STAT3, and their phosphorylated proteins were detected by Western blot; vertical axis, relative gray value; horizontal axis, group. (**B**) The expressions of AKT, PI3K, mTOR, 4EBP1, S6, and their phosphorylated proteins were detected by Western blot; vertical axis, relative gray value; horizontal axis, group. (**C**) The expressions of N-cadherin, MMP-2, p-FAK, t-FAK, β-catenin, and collagen I were detected by Western blot; vertical axis, relative gray value; horizontal axis, group. Data are shown as mean ± SEM (* *p* < 0.05; ** *p* < 0.01; ns indicates no significant difference (*p* ≥ 0.05)).

The results showed that the phosphorylation levels of Epidermal Growth Factor Receptor (EGFR) increased in HaCaT cells stimulated by DL-13 in vitro. Furthermore, the phosphorylation levels of extracellular regulated protein kinases (ERKs), Janus Kinase 2 (JAK2), and Signal Transducer and Activator of Transcription 3 (STAT3) signaling molecules downstream of the signaling pathway EGFR were also increased (Figure 5A). Meanwhile, the phosphorylation levels of Protein Kinase B (Akt), phosphatidylinositol-3 kinase (PI3K), and mammalian target of rapamycin (mTOR) were increased. Additionally, the phosphorylation levels of p-eIF4E-binding protein 1 (p-4EBP1) and p-ribosomal protein S6 (p-S6) signaling molecules downstream of the signaling pathway PI3K/AKT/mTOR were also increased (Figure 5B). At the same time, the expressions of marker proteins of epithelial–mesenchymal transition (EMT), such as Matrix Metallopeptidase 2 (MMP-2), p-Focal Adhesion Kinase (p-FAK), and N-cadherin (N-cad), were also increased in HaCaT cells stimulated by DL-13. It also increased the expression of collagen I, the main structural protein involved in skin wound healing (Figure 5C).

### 2.6. DL-13 Promoted the Reconstruction of Wounded Skin

**Figure 6:**
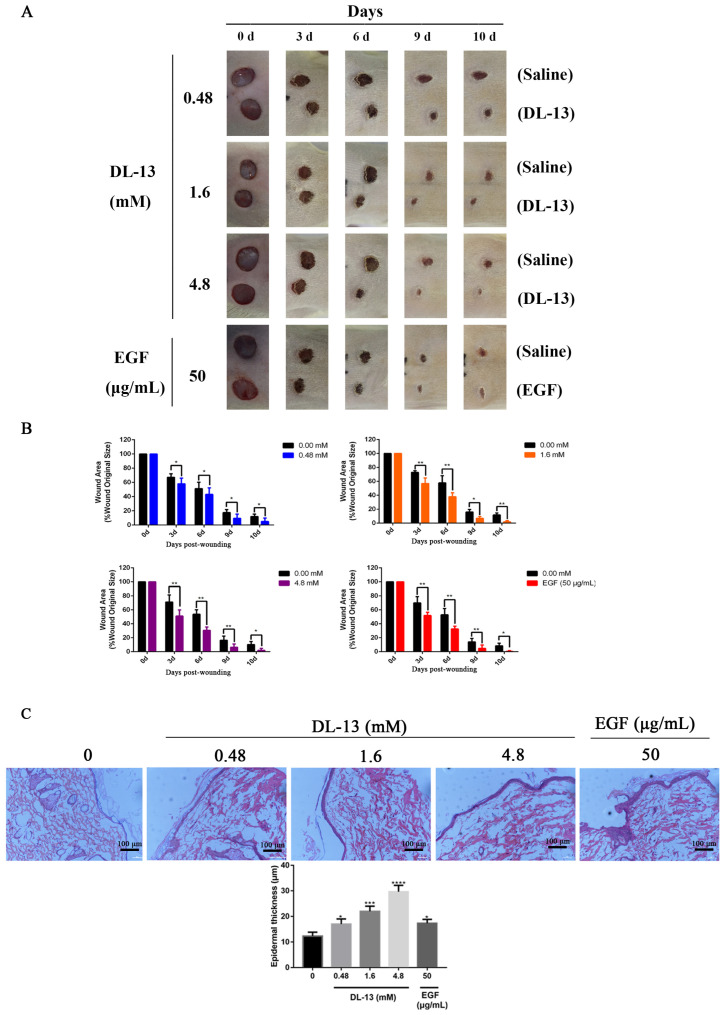
**Overall situation of wound healing in vivo in rats.** (**A**) Round wounds with a diameter of 1.0 cm were made on both sides of the rat’s body. The DL-13 solution was added to the wound surface in the experiment group, and an equal volume of deionized water was added in the control group. Each rat was photographed every other day. (**B**) Healing rate of wound in rat (n = 10). The area of the wound healed in every rat and the ratio to the initial total area were calculated by an image analyzer. Vertical axis, healing wound area expressed as % area; horizontal axis, time. (**C**) The results of hematoxylin and eosin staining (HE) of the wound skin (scale bar = 100 µm) and quantitative analysis of epidermal thickness at 10 d. Data are shown as mean ± SEM (* *p* < 0.05; ** *p* < 0.01; *** *p* < 0.001; **** *p* < 0.0001).

In order to study the effect of DL-13 on skin wound healing, a full-thickness skin wound model was made in the rats. The wound healing progress was analyzed at different time points. The results showed that the wound healing rate of the experimental group was significantly faster than that of the control group on the 3rd, 6th, 9th, and 10th days after injury (Figure 6A,B). Additionally, DL-13 treatment significantly increased the thickness of the epidermis in a concentration-dependent manner, with the 4.8 mM (4.8 mM ≈ 7598 μg/mL) concentration resulting in a notably thicker and structurally intact epidermis (Figure 6C).

## 3. Discussion

This study explores DL-13’s impact on skin wound healing in vivo and in vitro. Current wound healing therapies, while beneficial, often fall short. Techniques like negative pressure, oxygen therapy, and skin grafting improve wound closure but do not fully restore normal skin morphology [40]. Exogenous transforming growth factor-β (TGF-β) application has shown mixed results, leading to a shift towards alternative treatments [41]. Topical stem cell therapy has emerged as effective, but challenges in cell maintenance and protocol standardization persist [42]. Dressings, especially hydrogels and growth factors, are commonly used for wound care post-surgery. However, sustained growth factor application may have severe long-term effects [43]. DL-13 is found to enhance cutaneous wound repair by regulating key pathways such as JAK/STAT3 and PI3K/AKT synergistically.

Notably, DL-13 exhibits substantial clinical relevance and translational potential for wound care. Clinically, the current wound healing agents derived from *Periplaneta americana* are predominantly crude extracts (e.g., Kangfuxin) with complex compositions, leading to challenges in quality control, batch-to-batch variability, and unclear mechanisms of action [23,25]. In contrast, DL-13 is a pure, single-component peptide with well-defined structure (molecular weight 792 Da, purity > 95%), stable biological activity, and precise multi-pathway regulation (EGFR/PI3K-AKT/mTOR/ERK/MAPK/JAK-STAT3), addressing the key limitations of crude extracts. Its in vitro safety profile (no cytotoxicity at ≤50 μg/mL) and in vivo efficacy (dose-dependent acceleration of wound closure and epidermal regeneration) support its potential as a topical wound healing agent. For clinical application, DL-13 could be formulated into user-friendly dosage forms (e.g., hydrogels, films, or sprays) to enhance skin penetration and retention, suitable for treating acute wounds (e.g., surgical incisions, trauma) and potentially chronic wounds (e.g., diabetic foot ulcers) after validation in pathological models. Additionally, its peptide nature enables scalable synthesis via solid-phase peptide synthesis, facilitating large-scale production and standardization—critical prerequisites for clinical translation. Collectively, DL-13’s unique combination of structural clarity, targeted mechanism, safety, and manufacturability positions it as a promising candidate for developing next-generation wound repair drugs.

Keratinocyte proliferation is crucial for ensuring an adequate cell supply for migration and wound coverage [44]. Simultaneously, the directed migration of keratinocytes can promote faster healing of the skin space, which is very critical for wound epithelial formation [45]. Notably, our experimental data demonstrate that peptide DL-13 exhibits significant pro-proliferative and promigratory effects on human keratinocytes in vitro, indicating that it has biological activity in vitro to promote skin damage repair.

Numerous studies indicate that the inflammatory response significantly influences skin injury repair by affecting keratinocytes through interactions with specific inflammatory cell subsets and cytokines [46,47]. Our research supports the pro-repair role of peptide DL-13 in vitro by regulating *TNF-α* expression to reduce nitric oxide secretion by macrophages, thus inhibiting inflammation. However, evidence suggests a dual role of the inflammatory response in skin repair, which may explain why DL-13 did not effectively inhibit *IL-6* and *IL-1β* secretion in our study.

The JAK/STAT3 pathway is considered to be one of the most important signaling pathways in cells to transmit hormone, growth factor, and cytokine signals and participate in wound healing [48,49]. This is consistent with the dose-dependent upregulation of the protein expressions of p-JAK2 and p-STAT3 by peptide DL-13 in this study.

Additionally, transforming growth factor β1 (TGF-β1)-activated extracellular-regulated kinase 1/2 (ERK1/2) promotes skin repair by regulating pulp proteins and entering the nucleus to bind transcription factors such as c-fos and c-jun, thereby driving mitosis and differentiation [50]. Our finding that DL-13 upregulates p-ERK1/2 suggests it acts via the ERK/MAPK pathway.

EMT is crucial in wound healing, with EGF and TGF-β regulating partial EMT. EGFR-mediated EGF signaling controls Slug expression and keratinocyte activation [51]. Consistent with this, EGF activates EGFR and upregulates EMT markers. Similarly, DL-13 activates EGFR and increases EMT-related proteins (β-catenin, N-cadherin, MMP-2, p-FAK), indicating a shared mechanism with EGF. L-PRF is well documented to enhance epithelialization [14,15], and DL-13’s demonstrated modulation of EMT may independently yield analogous outcomes in cutaneous epithelialization. The upregulation of β-catenin, N-cadherin, MMP-2, and p-FAK, indicative of EMT induction by DL-13, suggests a potential mechanism for promoting keratinocyte migration and subsequent wound closure. Future studies could explore the potential synergistic effects of combining DL-13 with L-PRF, investigating whether DL-13-mediated EMT enhancement, coupled with the supportive extracellular matrix provided by L-PRF, could offer an improved therapeutic strategy for wound healing.

The PI3K/AKT/mTOR pathway also maintains skin homeostasis and supports wound repair [52]. Our results show that, after the action of DL-13, proteins related to the PI3K/AKT/mTOR signaling pathway, such as p-PI3K, p-AKT, and p-4EBP1, are significantly upregulated.

Studies have shown that both the ethanol extract of *Periplaneta americana* (PA) [53] and two proteoglycan complexes, PaPPc2 and PaPPc3 [54], can promote endothelial cell differentiation, angiogenesis, and collagen synthesis, thereby enhancing wound healing. Although clinically available drugs contain active extracts of PA, research investigating their active components remains relatively limited. Existing studies have primarily focused on crude active extracts, which still face challenges such as complex composition and unclear identification of specific bioactive constituents. The DL-13 protein identified in this study has a more well-defined composition, and research demonstrates that DL-13 can promote repair in vitro by activating the PI3K/AKT/mTOR signaling pathway while inducing EMT. Additionally, it can also act on the ERK/MAPK and JAK/STAT3 pathways regulated by EGFR. It has been proved that it can repair skin damage in rats.

The multi-pathway regulatory properties of DL-13 give it an advantage in wound repair, but its peptide nature may pose stability challenges. Subsequent research could explore combining it with biomaterials to enhance its stability and utilization efficiency. As previously demonstrated [14,15], L-PRF can accelerate wound epithelialization and reduce patient discomfort, suggesting its potential as a valuable adjunct to DL-13-based therapies. Future studies could investigate the synergistic effects of DL-13 and L-PRF on wound healing, with a focus on optimizing the delivery and application of these agents.

Despite the promising findings regarding the wound healing potential of DL-13, several limitations in this study must be acknowledged. First, although we compared the efficacy of DL-13 against a blank control and an EGF positive control, a scrambled peptide control was not included. Future studies should incorporate a scrambled sequence to definitively confirm that the observed biological activities are specific to the primary amino acid sequence of DL-13. Second, there is a notable difference in the concentration ranges used for in vitro (μg/mL level) and in vivo (mM level) experiments. The higher concentrations required for the animal model were selected to account for the complex wound environment and potential peptide degradation by tissue proteases [55]. The ~1000-fold difference is primarily attributed to the highly proteolytic in vivo wound microenvironment, which rapidly degrades exogenous peptides, and the physical barrier of the wound bed that necessitates a much higher concentration gradient than that in a controlled in vitro cell culture system. However, without direct pharmacokinetic and topical absorption data, this remains a hypothesis. Finally, while we demonstrated efficacy in an acute wound model, the long-term stability, pharmacokinetics, and potential off-target effects of DL-13 remain to be fully elucidated in future studies. Subsequent research will focus on analyzing the peptide’s stability in various physiological matrices and exploring formulation strategies to enhance its bioavailability for clinical application.

## 4. Conclusions

In summary, this study successfully isolated a novel peptide named DL-13 from *Periplaneta americana* L. The peptide demonstrates significant proliferative and migratory effects on HaCaT cells. Furthermore, DL-13 effectively suppresses the expressions of TNF-α and NO. Mechanistic investigations in HaCaT cells revealed that DL-13 exerts its biological functions by activating the PI3K/AKT/mTOR signaling pathway while inducing EMT, and it can modulate the ERK/MAPK and JAK/STAT3 pathways in vitro. In vivo experiments using rat models confirmed its remarkable wound healing properties. These findings collectively suggest that DL-13 holds great promise as a therapeutic agent for wound repair and regeneration, demonstrating considerable clinical potential.

## 5. Materials and Methods

### 5.1. Materials

Peptide compound DL-13 was isolated from the extract of *Periplaneta americana* L. using an ODS column (YMC C18, 5 μm, 4.6 × 250 mm), followed by purification through reversed-phase semipreparative HPLC. The purity of DL-13 was confirmed to be greater than 95% by analytical HPLC, and its molecular weight was determined by mass spectrometry. The experimental instrument is an Agilent 6210 LC/MSD TOF liquid chromatography–mass spectrometry system (Santa Clara, CA, USA). The mass range is 100–3000. The acquisition mode is positive ion mode. The gas temp is 300 °C; the drying gas is 11 L/min; the nebulizer is 35 psig; the Vcap is 2000 V; the fragmentor is 120 V; and the skimmer is 65 V. The analytical column is a YMC C8 chromatographic column (4.6 × 250 mm, 5 um). The chromatographic conditions are: mobile phase A (0.1% formic acid in water), mobile phase B (acetonitrile), gradient elution from 0 to 90 min (A:B = 95:5) to 10:90. HaCaT cells (Beyotime Biotechnology Co., Ltd., Shanghai, China). Raw 264.7 cells (TIB-71) were purchased from the American Culture Collection (ATCC, VA, USA). Mycoplasma testing was performed using Beyotime Biotechnology (C0297M), and the cells were negative. Cells were maintained in incomplete modified Eagle’s medium (DMEM) with 10% fetal bovine serum (FBS) (Gibco, CA, USA) and 1% of penicillin–streptomycin (Gibco, CA, USA) in a humidified 5% CO_2_ incubator at 37 °C.

### 5.2. Preparation of DL-13

The *Periplaneta americana* L. was crushed into coarse powder and then extracted three times with 3 times the volume of 80% (*v*/*v*) ethanol solution by maceration for 24 h each time. The extracted solutions were combined, evaporated under reduced pressure to a thick paste of approximately 1.5 L. Next, 10 times the volume of hot water (at 70 °C) was added, and the mixture was left to stand and separate for 12 h. The upper layer oil was discarded. The lower layer aqueous solution was concentrated and then applied as a sample onto a reversed-phase C-18 chromatographic column. Firstly, it was eluted with 30% methanol by volume for 6 column volumes and then eluted with 50% and 75% methanol-water solutions for 6 column volumes each. The eluate was analyzed by HPLC-MS for tracking and detection. The main peptide-containing fractions were collected, combined, evaporated under reduced pressure or dried under vacuum, to obtain the total peptide extract of the American cockroach. The total peptide extract was subjected to reversed-phase silica gel (ODS) column chromatography, using methanol–water as the eluent, with elution gradients of 10:90, 20:80, 30:70, 50:50, 70:30, and 90:10. Six main fractions Fr.A to Fr.F were obtained. Fraction Fr.D (3.2 g) was applied onto a Sephadex LH-20 chromatographic column (Cytiva, MA, USA) with water as the mobile phase, at a flow rate of 0.5 mL/min. After HPLC-MS analysis and detection, similar fractions were combined and collected to obtain 6 sub-fractions Fr.D1 to Fr.D6. Sub-fraction Fr.D3 was concentrated and dissolved in 5% volume fraction methanol and then separated and purified by reversed-phase preparative HPLC using a 50:50 acetonitrile–water eluent at a flow rate of 3 mL/min. The chromatographic peak with a retention time of 20.3 min was collected to obtain DL-13. In order to conduct subsequent experiments with a structure identical to that of DL-13 and higher purity, this study employed the conventional solid-phase synthesis method using an automatic peptide synthesis instrument. Through processes such as resin swelling, deprotection, washing, amino acid dissolution, amino acid activation, and condensation, the peptide DL-13 was synthesized.

### 5.3. Cell Viability

Cell viability was determined by the MTT method. Cell suspension was inoculated in a 96-well plate with 5000 cells in each well. The cells were pre-incubated in the incubator for 12 h to make the cells attach, and DL-13 was added according to the experimental group. After incubation for 24 and 48 h, MTT (1 mg/mL) (Sigma-Aldrich, St. Louis, MO, USA) was added for 45 min, then the culture medium was removed, and the formazan granules generated by live cells were dissolved in 100% DMSO and shaken for 10 min. The optical densities (ODs) at 550 and 630 nm were measured using a microplate reader (Aglient, Shanghai, China). The net absorbance (OD550–OD630) indicates the enzymatic activity of mitochondria and implies cell viability.

### 5.4. Cell Migration

HaCaT cells were planted into 6-well plates at 5 × 10^5^/well. The scratch was made by the 200 μL pipette tip on the bottom of the well vertically with the same force and washed by PBS twice. The DL-13 was added into the medium at 6.25 and 50 μg/mL. The blank control group was added with PBS. Finally, to quantify the cell migration rates at different time points (0, 24, and 48 h), the scratch-wound cell-free areas of each well were recorded using a microscope. The rate of wound closure was calculated using ImageJ software (https://imagej.net/downloads, accessed on 22 March 2024). The migration rate of the cell scratch experiment was calculated as follows: Migration rate (%) = (S_0_ − S_t_)/S_0_ × 100%, where S_0_ is the initial area of the cell scratch captured at 0 h, and S_t_ is the area of the cell scratch captured at 24 h and 48 h.

### 5.5. Levels of NO

The RAW 264.7 cells (2 × 10^6^/well) were seeded in a 6-well culture plate to ensure adhesion and then incubated with a vehicle or different concentrations of DL-13 for 24 h. To detect NO, the cells were also stimulated using lipopolysaccharide (LPS, 100 ng/mL, Sigma-Aldrich, MO, USA) from *Escherichia coli*. DXM as dexamethasone was the positive control. The supernatants were collected, and NO levels were analyzed using NO kits (Beyotime Biotechnology Co., Ltd., Shanghai, China).

### 5.6. Quantitative Real-Time PCR (qPCR)

The RAW264.7 cells (5 × 10^6^/well) were seeded in 6-well culture plates. After adhesion, the cells were cultured in serum-free medium and then incubated with vehicle or DL-13 for 24 h. To detect inflammatory cytokines, the cells were also stimulated using lipopolysaccharide (LPS, 100 ng/mL) from *Escherichia coli*. TRIzol reagent (Ambion Life Technologies, MA, USA) was used to isolate total RNA from the cells. After calculating the concentration of RNA, a cDNA synthesis kit (Takara, Kyoto, Japan) was used for reverse transcription. The prepared cDNA template and PCR kit were used to add samples according to the instructions, and then an instrument (CFX Connect™, BIO-RAD, CA, USA) was used for the qPCR reaction. Primers for inflammatory factors were designed based on the statistics from NCBI and PubMed. GAPDH was used as the internal reference.
*GAPDH*F: 5-GTCATTGAGAGCAATGCCAG-3.*GAPDH*R: 5-GTGTTCCTACCCCCAATGTG-3.*IL-6*F: 5-CTGACAATATGAATGTTGGG-3.*IL-6*R: 5-TCCAAGAAACCATCTGGCTAGG-3.*IL-1β*F: 5-GAGCCTGTGTTTCCTCCTTG-3.*IL-1β*R: 5-CAAGTGCAAGGCTATGACCA-3.*TNF-α*F: 5-GGGAGCAAAGGTTCAGTGAT-3.*TNF-α*R: 5-CCTGGCCTCTCTACCTTGTT-3.*Ki-67*F: 5-GAAGGAGAAGCAGCAGCAGATGAG-3.*Ki-67*R: 5-TGCTCCGCCGTCTTAAGGTAGG-3.

### 5.7. Western Blot

The HaCaT cells (5 × 10^6^/well) were seeded in 6-well culture plates. After adhesion, the cells were cultured in serum-free medium and incubated with vehicle or DL-13 at different concentrations. Harvested cells were lysed on ice using RIPA lysis buffer (Beyotime Biotechnology Co., Ltd., Shanghai, China) with protease and phosphatase inhibitors for 30 min. Then, the lysates were centrifuged for 10 min at 4 °C, 12,000× *g*. Supernatant was transferred to another tube and quantitated using a BCA protein assay kit (Beyotime Biotechnology Co., Ltd., Shanghai, China). Loading buffer (Thermo Fisher Scientific, MA, USA) was added to the supernatant, and samples were denatured at 100 °C for 10 min. Total protein extracts were subjected to SDS–polyacrylamide gel electrophoresis, transferred to PVDF membrane, and blocked with 5% BSA in TBST. Primary antibodies were incubated at 4 °C overnight, and secondary antibodies were incubated at room temperature for 1 h. Finally, signals were detected and images were taken. The primary antibodies we used are as follows: GAPDH, p-EGFR, Erk1/2, p-Erk1/2, p-JAK2, STAT3, p-STAT3, p-PI3K, p-AKT, AKT, p-mTOR, mTOR, p-4EBP1, 4EBP1, p-S6, N-cadherin, MMP-2, p-FAK, β-catenin, collagen I and S6 (1:1000, Cell Signaling Technology, MA, USA), detailed information about the antibodies is presented in Table S1.

### 5.8. Experimental Animals and Wound Model

Male rats (Jinan Pengyue Experimental Animal Breeding Co., LTD, Jinan, China), animal license number SYXK (Guangdong) 2017-0174) that were 6 weeks old and 100~120 g in weight were kept at 18–25 °C and 50% constant humidity (Jinan University Animal Laboratory Center). All the rats were allowed to access normal food and water freely. Rats were randomly assigned to one of four experimental groups, including different concentration treatment groups and the EGF group. Each group consisted of ten animals. The animal skin injury model was referenced by Moghadamtousi et al. and was further improved [56]. The hair on the backs of the rats was shaved carefully, and the skin was sterilized with 75% alcohol. The rats were anesthetized by intraperitoneal injection of 0.7% pentobarbital solution (Sigma, Michigan, USA) at 1.3 mL/kg, total 9.1 mg/kg. After the surgical site was prepared aseptically, two full-thickness skin wounds were created on the dorsal part via a 1 cm biopsy punch. The wounds in experimental rats were treated with 20 μL of DL-13 solution twice daily, while the control group was treated with an equal volume of deionized water. All the rats were photographed on the 3rd, 6th, 9th, and 10th days, and the diameter of the wound was measured using a vernier caliper, and the area of the wound was calculated using the formula for a circle. The wound area was analyzed by calculating the percentage of the current wound with respect to the initial wound area.

### 5.9. Hematoxylin and Eosin Staining

Rats were euthanized via carbon dioxide inhalation in accordance with the guidelines of the Laboratory Animal Welfare and Ethics Committee of Jinan University. The rat skin tissues were subjected to paraffin embedding, fixed with 10% neutral buffered formalin, and sectioned serially into 4 µm thick slices. Xylene and ethanol were used to deparaffinize the sectioned tissues in a dry oven at 60 °C. Tissues that had been deparaffinized were washed in the sink. Hematoxylin (Beyotime, Shanghai, China) and eosin (H&E) staining were performed for the sections, and all sections were observed and photographed under the microscope.

### 5.10. Statistical Analysis

All results were presented as the mean ± SEM. GraphPad Prism 5.0 was exclusively used for plotting and generating the figures. An independent sample *t*-test was used for comparison between two groups at the same time point, and two-way ANOVA was used for comparison between two groups at multiple time points. Following a significant omnibus test result from the one-way ANOVA, we performed the Tukey’s Honestly Significant Difference (HSD) post hoc test for all pairwise comparisons between groups. All statistical analyses presented in this study were performed using the software SPSS (version 22.0). A value of *p* < 0.05 was considered statistically significant; * *p* < 0.05, ** *p* < 0.01.

## Data Availability

All relevant data are available in the article.

## Supplementary Materials

The following supporting information can be downloaded at https://www.mdpi.com/article/10.3390/molecules31081355/s1. Table S1: Antibody table.

## Abbreviations

The following abbreviations are used in this manuscript:

HPLC	High-performance liquid chromatography
NO	Nitric oxide
TNF-α	Tumor necrosis factor-α
IL-6	Interleukin-6
IL-1β	Interleukin-1β
DMEM	Dulbecco’s modified Eagle medium
FBS	Fetal bovine serum
GAPDH	Glyceraldehyde-3-phosphate dehydrogenase
EGFR	Epidermal growth factor receptor
ERKs	Extracellular regulated protein kinases
JAK2	Janus kinase 2
STAT3	Signal transducer and activator of transcription 3
mTOR	Mammalian target of rapamycin
4EBP1	eIF4E-binding protein 1
OD	Optical density
PBS	Phosphate-buffered saline
PCR	Polymerase chain reaction
PI3K-Akt	Phosphatidylinositol 3-kinase–protein kinase B
PVDF	Polyvinylidene fluoride
RT-qPCR	Quantitative reverse transcription PCR
rpm	Revolutions per minute
TBS	Tris-buffered saline
WB	Western blot
