# Supplementary material for: The Effects and Molecular Mechanisms of a Peptide from Periplaneta americana L. in Skin Wound Healing
Source: Molecules. 2026 Apr 21;31(8):1355. doi: 10.3390/molecules31081355 (PMC13119250; doi:10.3390/molecules31081355)
Supplement: Supplementary file 1 [file molecules-31-01355-s001.zip › molecules-4114128-supplementary.pdf]

Supplementary table (Table S1)

Antibody	Host Species	Clonality	Catalog Number	Manufacturer
GAPDH	Rabbit	Monoclonal antibody	5174	Cell signaling Technology
β-Catenin	Rabbit	Monoclonal antibody	8480	Cell signaling Technology
p-EGFR	Rabbit	Monoclonal antibody	3777	Cell signaling Technology
Erk1/2	Rabbit	Monoclonal antibody	4695	Cell signaling Technology
p-Erk1/2	Rabbit	Polyclonal antibodies	9101	Cell signaling Technology
p-JAK2	Rabbit	Monoclonal antibody	3776	Cell signaling Technology
STAT3	Mouse	Monoclonal antibody	9139	Cell signaling Technology
p-STAT3	Rabbit	Monoclonal antibody	9145	Cell signaling Technology
p-PI3K	Rabbit	Polyclonal antibodies	4228	Cell signaling Technology
p-AKT	Rabbit	Monoclonal antibody	4060	Cell signaling Technology
AKT	Rabbit	Polyclonal antibodies	9272	Cell signaling Technology
p-mTOR	Rabbit	Polyclonal antibodies	2971	Cell signaling Technology
mTOR	Rabbit	Polyclonal antibodies	2972	Cell signaling Technology
p-4EBP1	Rabbit	Monoclonal antibody	2855	Cell signaling Technology
4EBP1	Rabbit	Monoclonal antibody	9644	Cell signaling Technology
p-S6	Rabbit	Monoclonal antibody	4858	Cell signaling Technology
S6	Rabbit	Monoclonal antibody	2217	Cell signaling Technology
N-cadherin	Rabbit	Polyclonal antibodies	4061	Cell signaling Technology
MMP-2	Rabbit	Polyclonal antibodies	4022	Cell signaling Technology
p-FAK	Rabbit	Polyclonal antibodies	3283	Cell signaling Technology
FAK	Rabbit	Polyclonal antibodies	3285	Cell signaling Technology
